# Crystal structure and Hirshfeld surface analysis of dibutyl 5,5′-(pentane-3,3-diyl)bis(1*H*-pyrrole-5-carboxylate)

**DOI:** 10.1107/S205698901900567X

**Published:** 2019-05-03

**Authors:** Haijing Wang, Zhenming Yin

**Affiliations:** a College of Chemistry, Tianjin Key Laboratory of Structure and Performance for Functional Molecules, Tianjin Normal University, Tianjin 300387, People’s Republic of China; b Key Laboratory of Inorganic–Organic Hybrid Functional Materials Chemistry (Tianjin Normal University), Ministry of Education, Tianjin 300387, People’s Republic of China

**Keywords:** dipyrro­methane-di­carboxyl­ate, crystal structure, hydrogen bonding

## Abstract

The mol­ecular structure of the title compound, C_23_H_34_N_2_O_4_, has *C*2 symmetry. In the crystal, inter­locked dimers are formed through quadruple N—H⋯O hydrogen bonds between pyrrole N—H groups and carbonyl O atoms.

## Chemical context   

Hydrogen-bonding inter­actions play an important role in the design of functional assemblies that exhibit a variety of properties and functions (Prins *et al.*, 2001 ▸; Steiner, 2002 ▸). Pyrrole-2-carboxyl­ate possesses one hydrogen-bond donor (N—H_pyrrole_) and one acceptor (C=O), which favour the formation of centrosymmetric dimers with pairs of N—H⋯O hydrogen bonds (Figueira *et al.*, 2015 ▸). The dimer motif is structurally similar to classic Watson–Crick nucleotide base-pairs. Calculations have revealed the dimer motif to be a robust supra­molecular synthon in crystal engineering (Dubis *et al.*, 2002 ▸). In previous work, we have shown a way to use the 2-carbonyl pyrrole dimer as a supra­molecular connector to construct hexa­gonal and grid architectures (Yin *et al.*, 2006 ▸). Here, we report the self-assembly of the title compound, *via* quadruple N—H⋯N hydrogen bonds.
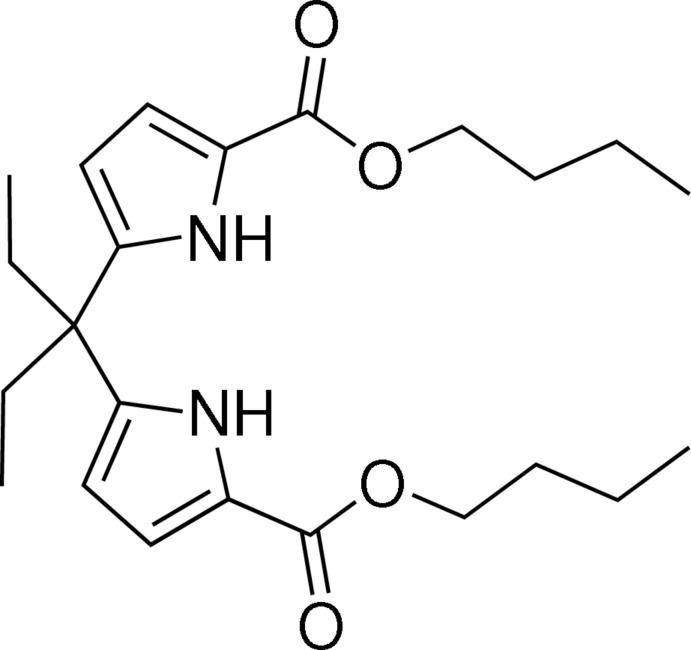


## Structural commentary   

**Figure 1:**
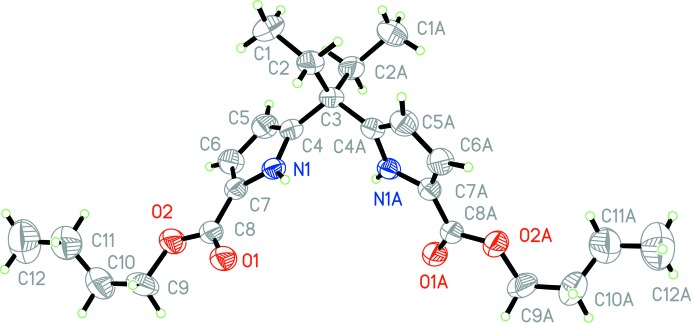
*ORTEP* diagram for the title compound, with displacement ellipsoids drawn at the 30% probability level. [Symmetry code: (A) *x*, 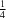 − *y*, 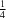 − *z*.]

The structure of the title compound is shown in Fig. 1 ▸. The asymmetric unit contains one half-mol­ecule as it possesses *C*2 symmetry. In the mol­ecule, the two pyrrole-2-carboxyl­ate groups are both in a *syn* conformation, with the carbonyl group arranged *syn* to its adjacent pyrrole NH group. The O1—C8—C7—N1 torsion angle is −8.2 (5)°. The dihedral angle between the pyrrole rings is 72.8 (2)°.

## Supra­molecular features   

**Figure 2:**
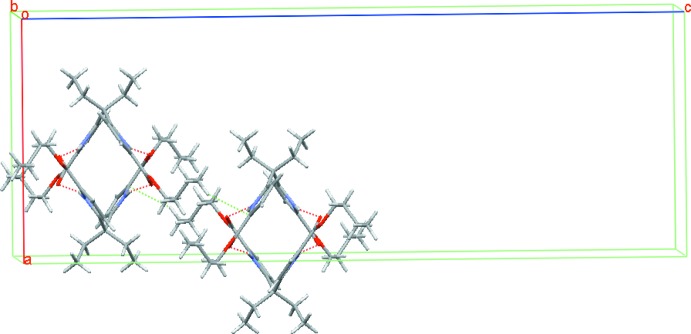
Part of the crystal packing showing mol­ecules linked by N—H⋯O hydrogen bonds (red dashed lines) and C—H⋯π contacts (green dashed lines). [Symmetry codes: (i) −*x* + 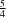, −*y* + 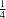, *z*; (ii) *x* + 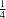, *y* + 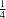 − *z*.]

Pairs of mol­ecules of the title compound form inter­locked dimers through four N1—H1⋯O1 hydrogen bonds between the pyrrole carbonyl oxygen atoms and pyrrole NH protons (Table 1 ▸, Fig. 2 ▸). This type of dimer has also been observed in our previous work (Yin *et al.*, 2007 ▸). The dimers are connected into a three-dimensional supra­molecular structure through C—H⋯π contacts (Table 1 ▸).

## Hirshfeld surface   

**Figure 3:**
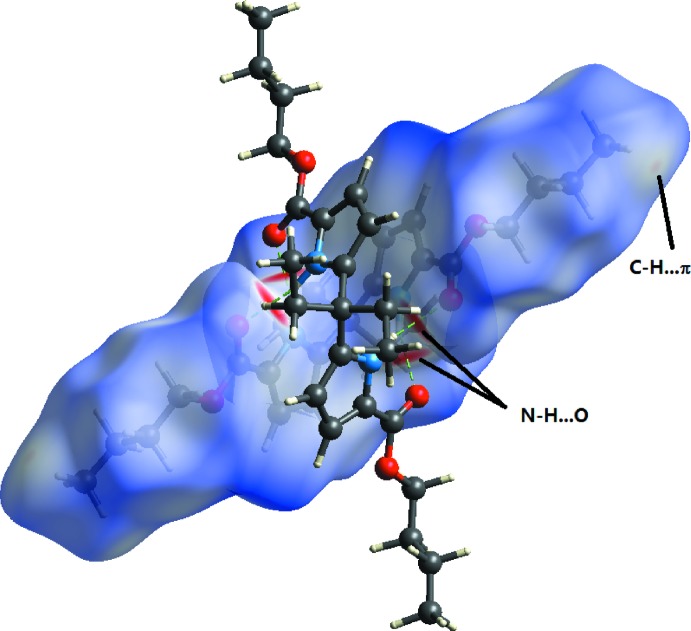
The Hirshfeld surface of the title compound mapped over *d*_norm_ in the range −0.486 to 1.895 a.u. The inter­molecular contacts can be seen in the red regions.

**Figure 4:**
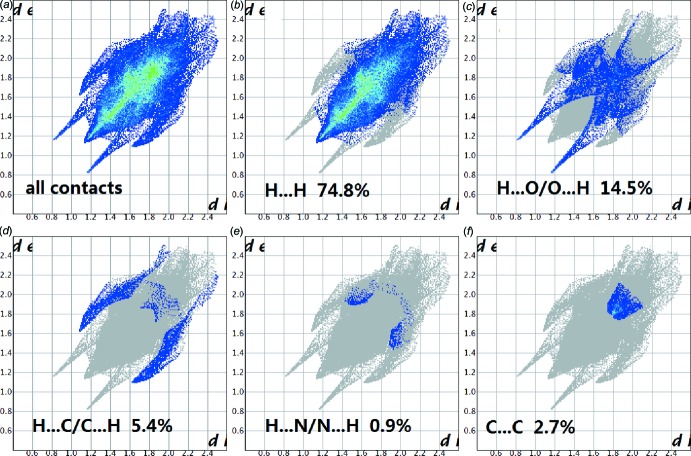
The two-dimensional fingerprint plots of title compound: (*a*) all contacts; (*b*) H⋯H, (*c*) H⋯O/O⋯H, (*d*) H⋯C/C⋯H, (*e*) H⋯N/N⋯H and (*f*) C⋯C.

A Hirshfeld surface analysis with *CrystalExplorer* (Turner *et al.*, 2017 ▸) was performed to give insights into the important inter­molecular inter­actions. These are normalized by van der Waals radii through a red–white–blue color scheme, where the red spots denote close contacts of mol­ecules. The three-dimensional *d*_norm_ surface of the title compound is shown in Fig. 3 ▸. The red points represent closer contacts and negative *d*_norm_ values on the surface corresponding to the N—H⋯O and C—H⋯π inter­actions mentioned above. The two-dimensional fingerprint plots in Fig. 4 ▸ shown the inter­molecular contacts and their percentage distributions on the Hirshfeld surface. H⋯H inter­actions (74.8%) are present as a major contributor while H⋯O/O⋯H (14.5%), H⋯C/C⋯H (5.4%), C⋯C (2.7%) and H⋯N/N⋯H (0.9%) contacts also give significant contributions to the Hirshfeld surface.

## Database survey   

A search in the Cambridge Structural Database (Groom *et al.*, 2016 ▸) returned over 60 entries for dipyrro­methane-1,9-dicarb­onyl derivatives, including seven entries whose supra­molecular structures feature inter­locked dimers (ILITAY, Love *et al.*, 2003 ▸; ODUMOQ,Yin *et al.*, 2007 ▸; PIRJAB, Xie *et al.*, 1994 ▸; NIQBAR01, Mahanta *et al.*, 2012 ▸; VACRID, Deliomeroglu *et al.*, 2016 ▸; PUJMAJ, Kim, 2010 ▸ and SAVDUQ, Uppal *et al.*, 2012 ▸). In the crystal of PUJMAJ (Kim, 2010 ▸), only one of the carbonyl groups is involved in hydrogen bonds with two pyrrole N—H groups.

## Synthesis and crystallization   

*n*-Butyl alcohol (370 mg, 5 mmol), 2,2′-ditrichlordi­pyrrole­methane (980 mg, 2 mmol) and tri­ethyl­amine (0.5 mL) were added to aceto­nitrile (20 mL), and then the mixture was refluxed for 2h. The solution was evaporated under reduced pressure and the residue was purified by column chromatography on silica gel (ethyl acetate/petroleum ether = 1:2), affording the title compound (white powder, 672 mg, 71%), m.p. = 388 K. ^1^H NMR (400 MHz, DMSO-*d*_6_); δ 0.64 (*t*, 6H, *J* = 7.2 Hz, –CH_3_), 0.90 (*t*, 6H, *J* = 7.2 Hz, –CH_3_), 1.31–1.41 (*m*, 4H, –CH_2_–), 1.58–1.65 (*m*, 4H, –CH_2_–), 2.15 (*q*, 4H, *J* = 7.2 Hz, Å –CH_2_–), 4.15 (q, 4H, *J* = 6.8 Hz, –CH_2_–), 5.97 (*s*, 2H, PyCH), 6.66 (*s*, 2H, PyCH), 11.22 (*s*, 2H, NH); HRMS (ESI) *m*/*z* calculated for C_23_H_34_N_2_O_4_, (*M* + H)^+^ 403.25186; found 403.25224. Crystals suitable for X-ray diffraction analysis were obtained by the slow evaporation of a CH_3_OH solution of the title compound.

## Refinement   

Crystal data, data collection and structure refinement details are summarized in Table 2 ▸. N—H hydrogen atoms were located from a difference-Fourier map and freely refined. Other H atoms were placed in difference calculated positions (C—H = 0.96 or 0.97 Å) and included in the final cycles of refinement using a riding model, with *U*_iso_(H) = 1.2*U*_eq_(C).

## Supplementary Material

Crystal structure: contains datablock(s) I. DOI: 10.1107/S205698901900567X/ff2158sup1.cif

Structure factors: contains datablock(s) I. DOI: 10.1107/S205698901900567X/ff2158Isup2.hkl

CCDC reference: 1912079

Additional supporting information:  crystallographic information; 3D view; checkCIF report

## Figures and Tables

**Table 1:** Hydrogen-bond geometry (Å, °) *Cg*1 is the centroid of the N1/C4–C7 ring.

*D*—H⋯*A*	*D*—H	H⋯*A*	*D*⋯*A*	*D*—H⋯*A*
N1—H1⋯O1^i^	0.86	2.12	2.962 (3)	165
C12—H12*C*⋯*Cg*1^ii^	0.96	3.21	3.944 (3)	135

Symmetry codes: (i) 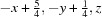; (ii) 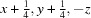.

**Table 2:** Experimental details

Crystal data	
Chemical formula	C_23_H_34_N_2_O_4_
*M*_r_	402.52
Crystal system, space group	Orthorhombic, *F* *d* *d* *d*
Temperature (K)	296
*a*, *b*, *c* (Å)	14.358 (6), 17.333 (7), 38.902 (19)
*V* (Å^3^)	9681 (7)
*Z*	16
Radiation type	Mo *K*α
μ (mm^−1^)	0.08
Crystal size (mm)	0.32 × 0.28 × 0.26

Data collection	
Diffractometer	Bruker SMART CCD area detector
Absorption correction	Multi-scan (*SADABS*; Bruker, 2001 ▸)
*T*_min_, *T*_max_	0.822, 1.000
No. of measured, independent and observed [*I* > 2σ(*I*)] reflections	11878, 2156, 1501
*R*_int_	0.031
(sin θ/λ)_max_ (Å^−1^)	0.595

Refinement	
*R*[*F* ^2^ > 2σ(*F* ^2^)], *wR*(*F* ^2^), *S*	0.081, 0.278, 1.05
No. of reflections	2156
No. of parameters	134
No. of restraints	2
H-atom treatment	H-atom parameters constrained
Δρ_max_, Δρ_min_ (e Å^−3^)	0.38, −0.34

Computer programs: *SMART* and *SAINT* (Bruker, 2001 ▸), *SHELXS* (Sheldrick, 2008 ▸), *SHELXL* (Sheldrick, 2015 ▸) and *OLEX2* (Dolomanov *et al.*, 2009 ▸).

## supplementary crystallographic information

### Crystal data

**Table:**

C_23_H_34_N_2_O_4_	*D*_x_ = 1.105 Mg m^−^^3^
*M**_r_* = 402.52	Mo *K*α radiation, λ = 0.71073 Å
Orthorhombic, *F**d**d**d*	Cell parameters from 3608 reflections
*a* = 14.358 (6) Å	θ = 2.4–23.4°
*b* = 17.333 (7) Å	µ = 0.08 mm^−^^1^
*c* = 38.902 (19) Å	*T* = 296 K
*V* = 9681 (7) Å^3^	Block, colourless
*Z* = 16	0.32 × 0.28 × 0.26 mm
*F*(000) = 3488	

### Data collection

**Table:**

Bruker SMART CCD area detector diffractometer	1501 reflections with *I* > 2σ(*I*)
Graphite monochromator	*R*_int_ = 0.031
phi and ω scans	θ_max_ = 25.0°, θ_min_ = 1.9°
Absorption correction: multi-scan (SADABS; Bruker, 2001)	*h* = −17→16
*T*_min_ = 0.822, *T*_max_ = 1.000	*k* = −20→18
11878 measured reflections	*l* = −46→44
2156 independent reflections	

### Refinement

**Table:**

Refinement on *F*^2^	Primary atom site location: structure-invariant direct methods
Least-squares matrix: full	Hydrogen site location: inferred from neighbouring sites
*R*[*F*^2^ > 2σ(*F*^2^)] = 0.081	H-atom parameters constrained
*wR*(*F*^2^) = 0.278	*w* = 1/[σ^2^(*F*_o_^2^) + (0.1517*P*)^2^ + 16.1858*P*] where *P* = (*F*_o_^2^ + 2*F*_c_^2^)/3
*S* = 1.05	(Δ/σ)_max_ = 0.002
2156 reflections	Δρ_max_ = 0.38 e Å^−^^3^
134 parameters	Δρ_min_ = −0.34 e Å^−^^3^
2 restraints	

### Special details

**Table:**

Geometry. All esds (except the esd in the dihedral angle between two l.s. planes) are estimated using the full covariance matrix. The cell esds are taken into account individually in the estimation of esds in distances, angles and torsion angles; correlations between esds in cell parameters are only used when they are defined by crystal symmetry. An approximate (isotropic) treatment of cell esds is used for estimating esds involving l.s. planes.

### Fractional atomic coordinates and isotropic or equivalent isotropic displacement parameters (Å^2^)

**Table:**

	*x*	*y*	*z*	*U*_iso_*/*U*_eq_	
O1	0.68341 (16)	0.20703 (14)	0.05576 (7)	0.0943 (8)	
O2	0.65735 (17)	0.33467 (14)	0.05760 (8)	0.1078 (10)	
N1	0.51592 (15)	0.18458 (13)	0.09406 (6)	0.0673 (7)	
H1	0.5364	0.1412	0.0865	0.081*	
C1	0.2522 (3)	0.1657 (3)	0.08110 (13)	0.1320 (17)	
H1A	0.2131	0.1822	0.0997	0.198*	
H1B	0.2142	0.1459	0.0628	0.198*	
H1C	0.2880	0.2087	0.0729	0.198*	
C2	0.3186 (2)	0.1018 (2)	0.09382 (9)	0.0941 (11)	
H2A	0.3591	0.0869	0.0750	0.113*	
H2B	0.2819	0.0570	0.1001	0.113*	
C3	0.3798 (3)	0.1250	0.1250	0.0756 (11)	
C4	0.44025 (19)	0.19324 (17)	0.11502 (7)	0.0704 (8)	
C5	0.4317 (2)	0.27091 (19)	0.12144 (10)	0.0906 (10)	
H5	0.3864	0.2937	0.1352	0.109*	
C6	0.5026 (3)	0.31011 (19)	0.10378 (10)	0.0919 (10)	
H6	0.5126	0.3631	0.1036	0.110*	
C7	0.5546 (2)	0.25550 (17)	0.08689 (8)	0.0749 (8)	
C8	0.6375 (2)	0.26101 (19)	0.06550 (9)	0.0810 (9)	
C9	0.7383 (3)	0.3480 (3)	0.03496 (16)	0.141 (2)	
H9A	0.7953	0.3356	0.0471	0.169*	
H9B	0.7342	0.3149	0.0149	0.169*	
C10	0.7401 (4)	0.4274 (4)	0.02437 (19)	0.164 (2)	
H10A	0.7967	0.4359	0.0113	0.197*	
H10B	0.7440	0.4592	0.0448	0.197*	
C11	0.6604 (5)	0.4549 (4)	0.0035 (2)	0.193 (3)	
H11A	0.6551	0.4210	−0.0162	0.232*	
H11B	0.6045	0.4478	0.0172	0.232*	
C12	0.6597 (7)	0.5314 (4)	−0.0088 (2)	0.211 (4)	
H12A	0.6611	0.5666	0.0102	0.316*	
H12B	0.6042	0.5399	−0.0221	0.316*	
H12C	0.7133	0.5398	−0.0231	0.316*	

### Atomic displacement parameters (Å^2^)

**Table:**

	*U*^11^	*U*^22^	*U*^33^	*U*^12^	*U*^13^	*U*^23^
O1	0.0756 (14)	0.0847 (16)	0.1227 (19)	0.0097 (12)	0.0142 (12)	0.0162 (13)
O2	0.0842 (17)	0.0826 (16)	0.157 (2)	0.0004 (12)	0.0174 (15)	0.0302 (14)
N1	0.0571 (13)	0.0653 (13)	0.0796 (15)	0.0047 (10)	−0.0016 (10)	0.0003 (11)
C1	0.089 (3)	0.169 (4)	0.138 (4)	−0.001 (3)	−0.041 (3)	0.027 (3)
C2	0.072 (2)	0.116 (3)	0.095 (2)	−0.0173 (18)	−0.0137 (16)	0.0109 (19)
C3	0.053 (2)	0.090 (3)	0.083 (2)	0.000	0.000	0.006 (2)
C4	0.0570 (15)	0.0767 (18)	0.0775 (17)	0.0077 (13)	−0.0020 (12)	0.0027 (14)
C5	0.084 (2)	0.083 (2)	0.104 (2)	0.0180 (17)	0.0106 (18)	−0.0065 (18)
C6	0.088 (2)	0.0640 (18)	0.124 (3)	0.0049 (15)	0.005 (2)	0.0003 (18)
C7	0.0648 (17)	0.0672 (17)	0.093 (2)	0.0018 (13)	−0.0053 (15)	0.0099 (14)
C8	0.0659 (18)	0.0748 (19)	0.102 (2)	0.0011 (15)	−0.0014 (16)	0.0153 (16)
C9	0.075 (2)	0.129 (3)	0.219 (6)	−0.003 (2)	0.039 (3)	0.043 (4)
C10	0.136 (4)	0.149 (4)	0.206 (6)	−0.016 (4)	0.033 (4)	0.071 (4)
C11	0.156 (6)	0.205 (6)	0.219 (7)	0.028 (5)	0.033 (5)	0.085 (6)
C12	0.276 (11)	0.175 (5)	0.182 (6)	0.040 (7)	0.051 (6)	0.036 (5)

### Geometric parameters (Å, º)

**Table:**

O1—C8	1.205 (4)	C5—H5	0.9300
O2—C8	1.344 (4)	C5—C6	1.404 (5)
O2—C9	1.477 (5)	C6—H6	0.9300
N1—H1	0.8600	C6—C7	1.372 (5)
N1—C4	1.367 (4)	C7—C8	1.456 (5)
N1—C7	1.377 (4)	C9—H9A	0.9700
C1—H1A	0.9600	C9—H9B	0.9700
C1—H1B	0.9600	C9—C10	1.436 (7)
C1—H1C	0.9600	C10—H10A	0.9700
C1—C2	1.542 (6)	C10—H10B	0.9700
C2—H2A	0.9700	C10—C11	1.481 (9)
C2—H2B	0.9700	C11—H11A	0.9700
C2—C3	1.551 (4)	C11—H11B	0.9700
C3—C2^i^	1.551 (4)	C11—C12	1.412 (8)
C3—C4	1.518 (4)	C12—H12A	0.9600
C3—C4^i^	1.518 (4)	C12—H12B	0.9600
C4—C5	1.375 (4)	C12—H12C	0.9600
			
C8—O2—C9	116.9 (3)	N1—C7—C8	120.3 (3)
C4—N1—H1	125.0	C6—C7—N1	107.4 (3)
C4—N1—C7	110.1 (2)	C6—C7—C8	132.3 (3)
C7—N1—H1	125.0	O1—C8—O2	123.4 (3)
H1A—C1—H1B	109.5	O1—C8—C7	125.1 (3)
H1A—C1—H1C	109.5	O2—C8—C7	111.5 (3)
H1B—C1—H1C	109.5	O2—C9—H9A	109.8
C2—C1—H1A	109.5	O2—C9—H9B	109.8
C2—C1—H1B	109.5	H9A—C9—H9B	108.2
C2—C1—H1C	109.5	C10—C9—O2	109.6 (4)
C1—C2—H2A	108.6	C10—C9—H9A	109.8
C1—C2—H2B	108.6	C10—C9—H9B	109.8
C1—C2—C3	114.5 (3)	C9—C10—H10A	108.1
H2A—C2—H2B	107.6	C9—C10—H10B	108.1
C3—C2—H2A	108.6	C9—C10—C11	116.8 (6)
C3—C2—H2B	108.6	H10A—C10—H10B	107.3
C2—C3—C2^i^	111.0 (4)	C11—C10—H10A	108.1
C4^i^—C3—C2^i^	109.02 (17)	C11—C10—H10B	108.1
C4^i^—C3—C2	108.81 (18)	C10—C11—H11A	107.4
C4—C3—C2^i^	108.81 (18)	C10—C11—H11B	107.4
C4—C3—C2	109.02 (17)	H11A—C11—H11B	106.9
C4^i^—C3—C4	110.2 (3)	C12—C11—C10	119.7 (8)
N1—C4—C3	121.5 (2)	C12—C11—H11A	107.4
N1—C4—C5	106.7 (3)	C12—C11—H11B	107.4
C5—C4—C3	131.7 (3)	C11—C12—H12A	109.5
C4—C5—H5	125.7	C11—C12—H12B	109.5
C4—C5—C6	108.7 (3)	C11—C12—H12C	109.5
C6—C5—H5	125.7	H12A—C12—H12B	109.5
C5—C6—H6	126.4	H12A—C12—H12C	109.5
C7—C6—C5	107.1 (3)	H12B—C12—H12C	109.5
C7—C6—H6	126.4		
			
O2—C9—C10—C11	−62.9 (8)	C4^i^—C3—C4—N1	44.80 (19)
N1—C4—C5—C6	0.7 (4)	C4^i^—C3—C4—C5	−140.2 (4)
N1—C7—C8—O1	−8.2 (5)	C4—C5—C6—C7	−0.5 (4)
N1—C7—C8—O2	171.6 (3)	C5—C6—C7—N1	0.1 (4)
C1—C2—C3—C2^i^	59.3 (3)	C5—C6—C7—C8	−178.0 (3)
C1—C2—C3—C4	−60.5 (4)	C6—C7—C8—O1	169.8 (4)
C1—C2—C3—C4^i^	179.2 (3)	C6—C7—C8—O2	−10.4 (5)
C2—C3—C4—N1	−74.5 (3)	C7—N1—C4—C3	175.5 (2)
C2^i^—C3—C4—N1	164.3 (3)	C7—N1—C4—C5	−0.6 (3)
C2—C3—C4—C5	100.5 (4)	C8—O2—C9—C10	170.1 (5)
C2^i^—C3—C4—C5	−20.7 (4)	C9—O2—C8—O1	1.8 (6)
C3—C4—C5—C6	−174.9 (3)	C9—O2—C8—C7	−178.0 (4)
C4—N1—C7—C6	0.3 (3)	C9—C10—C11—C12	−177.5 (6)
C4—N1—C7—C8	178.7 (3)		

Symmetry code: (i) *x*, −*y*+1/4, −*z*+1/4.

### Hydrogen-bond geometry (Å, º)

*Cg*1 is the centroid of the N1/C4–C7 ring.

**Table:**

*D*—H···*A*	*D*—H	H···*A*	*D*···*A*	*D*—H···*A*
N1—H1···O1^ii^	0.86	2.12	2.962 (3)	165
C12—H12*C*···*Cg*1^iii^	0.96	3.21	3.944 (3)	135

Symmetry codes: (ii) −*x*+5/4, −*y*+1/4, *z*; (iii) *x*+1/4, *y*+1/4, −*z*.
